# Targeting glucose control in preterm infants: pilot studies of continuous glucose monitoring

**DOI:** 10.1136/archdischild-2018-314814

**Published:** 2018-09-19

**Authors:** Lynn Thomson, Daniela Elleri, Simon Bond, James Howlett, David B Dunger, Kathryn Beardsall

**Affiliations:** 1 Department of Paediatrics, University of Cambridge, Cambridge, UK; 2 Neonatal Unit, Cambridge University Hospitals NHS Foundation Trust, Cambridge, UK; 3 Cambridge Clinical Trials Unit, Cambridge University Hospitals NHS Foundation Trust, Cambridge, UK; 4 MRC Biostatistics Unit, University of Cambridge, Institute of Public Health, Cambridge, UK; 5 Wellcome Trust MRC Institute of Metabolic Science, University of Cambridge, Addenbrooke’s Hospital NHS Trust, Cambridge, UK

**Keywords:** glucose, hyperglycaemia, hypoglycaemia, continuous glucose monitoring

## Abstract

**Objective:**

Hyperglycaemia is common in very preterm infants and is associated with adverse outcomes. Preventing hyperglycaemia without increasing the risk of hypoglycaemia is difficult. Real time tracking with continuous glucose monitors (CGM) may improve glucose control. We assessed the feasibility and safety of CGM to target glucose control in preterm infants, to inform a randomised controlled trial (RCT).

**Design:**

We performed a single centre study in very preterm infants during the first week of life. Accuracy was assessed by comparison of CGM with blood glucose levels (n=20 infants). In a separate pilot study of efficacy (n=20), real-time CGM combined with a paper guideline to target glucose control (2.6–10 mmol/L) was compared with standard neonatal care (masked CGM). Questionnaires were used to assess staff acceptability.

**Results:**

No concerns were raised about infection or skin integrity at sensor site. The sensor performed well compared with point-of-care blood glucose measurements, mean bias of −0.27 (95% CI −0.35 to −0.19). Per cent time in target range (sensor glucose 2.6–10 mmol/L) was greater with CGM than POC (77% vs 59%, respectively) and per cent time sensor glucose >10 mmol/L was less with CGM than POC (24% vs 40%, respectively). The CGM also detected clinically unsuspected episodes of hypoglycaemia. Staff reported that the use of the CGM positively improved clinical care.

**Conclusions:**

This study suggests that CGM has sufficient accuracy and utility in preterm infants to warrant formal testing in a RCT.

**What is already known on this topic?**

- Glucose dysregulation is common in neonatal intensive care and management may be challenging.
- Hyperglycaemia and hypoglycaemia are associated with adverse clinical outcomes.
- Use of continuous glucose monitoring (CGM) has been limited by controversy regarding optimal targets and impact on staff workload.

**What this study adds?**

- CGM can support the use of insulin for glucose control in preterm infants and highlight clinically silent hypoglycaemia.
- Staff reported that CGM use improved clinical care.
- Accuracy and utility of CGM in preterm infants are sufficient to warrant multicentre trials.

## Introduction

Continuous glucose monitoring (CGM) was first developed to support glucose control in patients with diabetes mellitus.^1 2^ It has also been used in intensive care for safer targeting of glucose levels, predominantly in adults,^2–6^ but its role remains controversial.^7^ Extremely preterm infants have a high prevalence of hyperglycaemia and hypoglycaemia,^8–11^ which are associated with adverse outcomes.^8 10 12^ Glucose monitoring in neonatal intensive care is infrequent due to the desire for minimal handling and limitation of blood sampling.^13^ Hyperglycaemia is often difficult to manage without either compromising nutrition or risking inadvertent hypoglycaemia following the use of insulin.^14 15^

Earlier CGM models have been used in the preterm population to collect data masked to the clinical team and have shown sensors to be well tolerated.^16–19^ The challenges to using the CGM to target glucose control are related to clinical confidence in the accuracy of the device,^20 21^ the wide variation in insulin sensitivity between babies and concerns about increased workload for nursing staff. This study aimed to assess the feasibility of CGM to support targeting of glucose control in preterm infants, to inform the design of a future randomised controlled trial.

## Design

We performed a single centre study at the University of Cambridge Addenbrookes Hospital NHS Trust, where ethics committee and trust approval had been obtained. There were two phases: (1) an observational study to assess the accuracy of the CGM and (2) a pilot study in which babies were randomised to real-time CGM or standard care. Inclusion criteria were birth weight <1200 g, age <48 hours and written informed parental consent, and exclusion criteria, any baby with a major congenital malformation, any underlying metabolic disorder or if mothers had diabetes mellitus. Babies remained in the study until 7 days of age.

## Methods

### Continuous glucose monitoring

**Figure 1:**
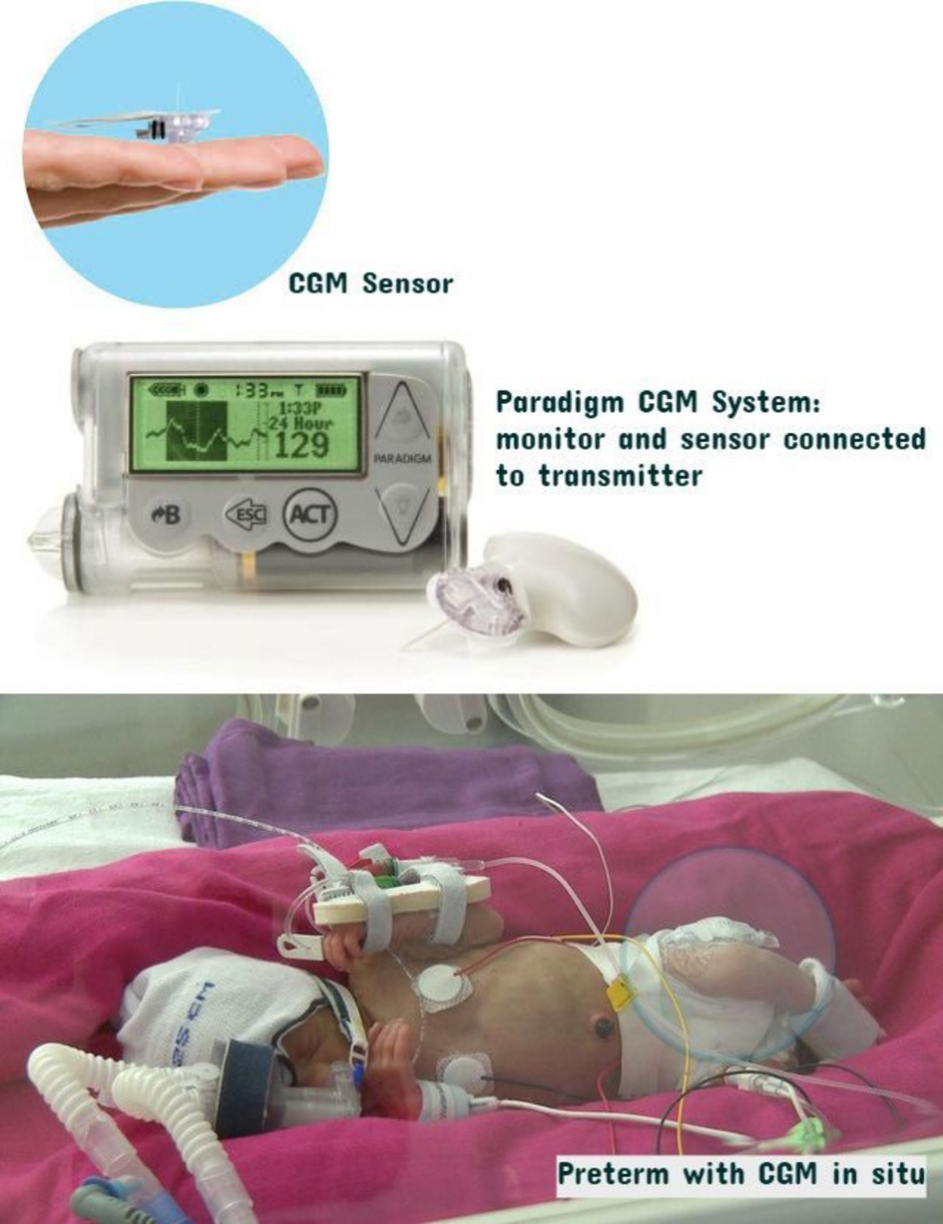
Continuous glucose monitoring (CGM) System demonstrating: i. Enlite sensor, ii. Paradigm Veo and MiniLink Transmitter attached to a sensor and iii. preterm infant with device in situ.

Real-time CGM was performed using the Paradigm Veo (Medtronic, Watford UK; figure 1), which was calibrated at least twice daily using blood glucose (BG) levels measured on the Statstrip meter (Nova Biomedical). In the control group, masked data were collected using the Ipro 2 (Medtronic, Watford, UK) which was calibrated at data download using BG levels taken for clinical management (minimum of two in 24 hours).

### BG monitoring

BG levels were measured using a combination of arterial, venous or capillary samples and tested on the blood gas analyser (Cobas b221; Roche Diagnostics, UK), and the Nova StatStrip (Nova Biomedical, Massachusetts, USA).

#### Accuracy of the CGM

The Paradigm Veo real-time CGM was assessed for accuracy. It was calibrated at least twice daily using a BG measured on the point-of care Statstrip meter. The Statstrip meter was chosen because it has been validated for accuracy in the newborn and intensive care settings. Prespecified comparative analyses were based on any glucose levels that were recorded within 5 min of each other. Median relative difference was calculated as the percentage difference between the two measures. Absolute differences were determined at each time point in terms of compliance with ISO2003 and ISO2013 standards.^22 23^ Bland-Altman analyses was used for assessment of error between glucose measurements, and error grid plots to explore potential clinical impact.

#### Pilot study

Babies were randomised 1:1 to either control (standard care with masked CGM data collection) or to intervention with glucose control supported by use of real-time CGM monitoring along with a specifically designed paper guideline. Randomisation using a simple computer randomisation programme that included minimisation of differences in gestational age and birth weight took place within 48 hours of birth.^24^

##### Intervention: continuous glucose monitoring with paper guideline

Enlite sensors were linked to Paradigm Veo. This allowed real-time viewing of sensor glucose (SG) data, which were used in conjunction with the paper guideline to support clinical management (online supplementary figure). The latter provided simple guidance and was not a rigid algorithm and had not undergone formal in silico testing. The nurses recorded the SG value alongside standard hourly clinical observation, using it to guide the need for BG testing. The guideline prompted review and intervention based on both absolute glucose levels and change.

10.1136/archdischild-2018-314814.supp1 Supplementary file 1

##### Control: standard care

Enlite sensors were linked to an Ipro 2 to collect data prospectively but blinded to the clinical team. Standard care aimed to target glucose levels between 2.6 and 10 mmol/L by reduction of dextrose intake or use of sliding scale insulin infusion at the discretion of the clinical team. Sliding scale insulin was considered if BG levels were >10 mmol/L on more than two occasions. The masked CGM data were downloaded on day 7, at the end of the study period.

### Analyses

Predefined efficacy outcomes included time in target (per cent time SG 2.6–10 mmol/L), time in target (SG 4–8 mmol/L), prevalence of hyperglycaemia (per cent time SG >10 mmol/L) and severe hyperglycaemia (per cent time SG >15 mmol/L). Data regarding glucose levels, clinical condition and nutritional intake, as well as insulin use, were collected prospectively. Results are expressed as mean ±SD, median (IQR) or frequencies (percentages) as appropriate. Safety outcomes were defined as prevalence of hypoglycaemia (per cent time SG <2.6 mmol/L) and any single BG <2.6 mmol/L and/or more than six SG readings <2.6 mmol/L (ie, >30 min).

### Staff perspective on clinical care

The clinical care team were invited in both studies to complete a comments sheet daily for immediate feedback as well as an anonymised questionnaire for summative review. The questionnaires explored initial expectations as well as experiences of using the CGM. These questionnaires were developed in collaboration with nurses on the unit to ensure that questions were easy to understand and relevant to the individuals concerned.

## Results

No concerns were raised about the sensor site in terms of skin integrity, infection or inflammation in any of the babies. Twenty-one babies were recruited to the accuracy study (one was withdrawn due to failure of sensor insertion). Twenty-three babies were recruited to the pilot study. No CGM data were found at the time of data download in two control babies, and one baby in the intervention group died of a massive pulmonary haemorrhage, within 24 hours of birth. These babies were excluded from the analyses. Baseline demographic details of the babies with CGM data are shown in table 1. The babies in each study group appear comparable. In the pilot study, a larger per cent of babies in the intervention group had prolonged rupture of membranes or chorioamnionitis. One baby in the intervention group required a sensor to be replaced due to loss of connectivity between the sensor and the monitor.

**Table 1:** Demographic details and nutritional intake of infants in all study groups

	Accuracy study (n=20)	Intervention study control (n=10)	Intervention study real-time CGM (n=10)
Gestational age at birth (weeks)	26.14 (1.9)	27.96 (2.1)	27.5 (2.8)
Birth weight (g)	809 (156)	901 (144)	823 (282)
Sex (male:female)	10:10	6:4	5:5
Antenatal variables			
Antenatal steroids	19 (95%)	10 (100%)	9 (90%)
Maternal smoking	5 (25%)	1 (10%)	2 (20%)
Chorioamnionitis	1 (5%)	0 (0%)	3 (30%)
PROM	6 (30%)	2 (20%)	4 (40%)
Hypertension	1 (5%)	2 (20%)	1 (10%)
Nutritional intake			
Mean dextrose infused (mg/kg/min)		6.10 (2.2)	7.73 (2.6)
Mean protein infused (g/kg/day)		2.98 (1.15–3.67)	3.38 (2.58–3.97)
Mean lipid infused (g/kg/day)		1.80 (0.63–2.22)	1.44 (1.10–2.19)
Mean oral feeds (mL/kg/hour)		0.01 (0.00–0.03)	0.01 (0.00–0.03)

Antenatal factors associated with hyperglycaemia, hypoglycaemia and postnatal nutrition appear comparable between study groups.

PROM is >24 hours.

Data are presented as mean±SD, number (%) or median (IQR) as appropriate.

CGM, continuous glucose monitoring; PROM, prolonged rupture of membrane.

### Accuracy of CGM

**Figure 2:**
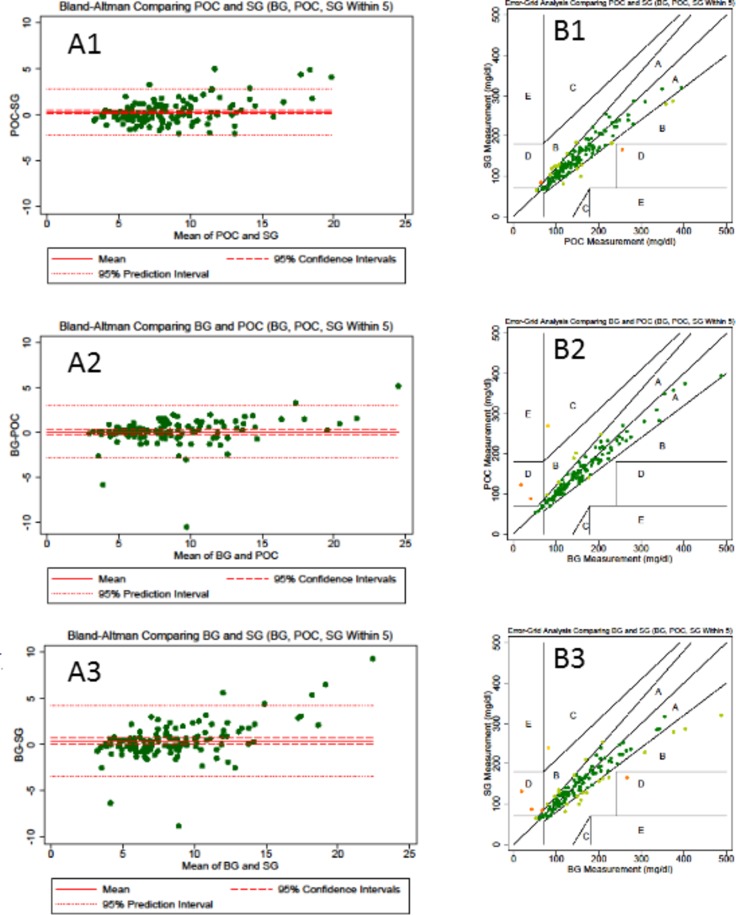
Bland-Altman and error grid plot. A1: Bland-Altman comparison of CGM SG with Statstrip meter (Nova Biomedical). A2: Bland Altman comparison of Statstrip meter (Nova Biomedical) with blood gas (Cobas b221, Roche Diagnostics) blood glucose (BG) values. A3: Bland-Altman comparison of CGM SG with blood gas (Cobas b221) BG values. B1: Error grid plot comparing CGM SG with Statstrip meter (Nova Biomedical) or blood gas (Cobas b221) BG values. B2: Error grid plot comparing point-of-care Statstrip meter (Nova Biomedical) with blood gas (Cobas b221) BG values. B3: Error grid plot comparing CGM SG with blood gas (Cobas b221) BG values. The reference lines show the estimate of the mean diﬀerence, 95% CIs around the mean estimate and the predictive interval which indicate the region in which a new observation would expect to be observed with 95% CI. BG, blood glucose level; POC, point-of-care blood glucose; SG, sensor glucose.

Comparative data providing more than two glucose measurements were available at 247 time points. Performance in relation to the ISO2003 and ISO2013 standards is provided in table 2. Bland-Altman analyses provided a mean bias between point of care and CGM of −0.27 (95% CI −0.35 to −0.19) (figure 2). Error grid analyses (comparing SG with either BG methodology) demonstrated that 98% of values lay within area A or B (figure 2).

**Table 2:** Comparison of sensor glucose measurements with point-of-care glucose values and blood glucose values

	Criteria	Blood glucose	Point of care
MARD	% difference between SG and reference (SG-reference)/reference × 100	11.2%	9.7%
ISO2003*		84%	90%
ISO2013†		73%	78%

ISO2003, ISO2013.^22 23^

CGM systems have previously been reported to show MARD results between 10% and 20% in adults and paediatrics.

*System accuracy standards of 2003 (ISO: 15197:2003) states that 95% of blood glucose results should be within ±0.83 mmol/L of laboratory results at concentrations <4.2 mmol/L or within ±20% of laboratory results at concentrations of >4.2 mmol/L.

†System accuracy standards 2013 (ISO: 15197:2013) require that 95% of blood glucose results should be within ±0.83 mmol/L of laboratory results at concentrations of <5.6 mmol/L or within ±20% of laboratory results at concentrations of ≤5.6 mmol/L.

CGM, continuous glucose monitoring; MARD, mean absolute relative difference; SG, sensor glucose.

### Pilot study

**Figure 3:**
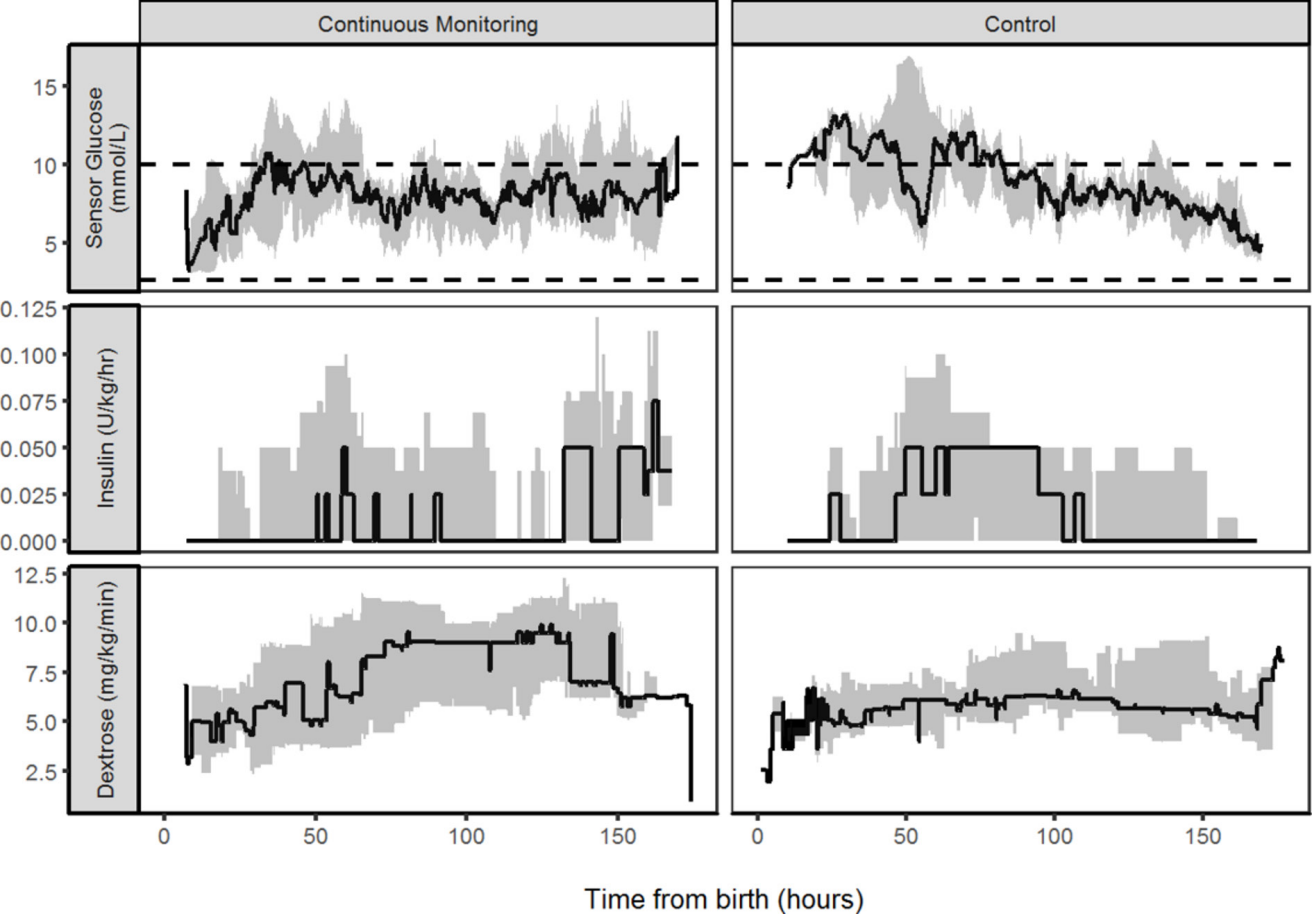
Comparison of sensor glucose and insulin infusion during the first week of life. Median (IQR) of sensor glucose and insulin infused in babies with real-time continuous glucose monitoring and standard care. The target glucose range of 2.6 to 10.0 mmol/L is denoted by horizontal lines.

In the 20 babies recruited to the efficacy study, the median (range) length of glucose data collected in control and intervention groups were 142.5 (90.25–148.17) hours and 140.5 (89.33–143.75) hours, respectively. Data demonstrating the per cent time within different target thresholds are provided in table 3 and figure 3. There was wide variability in glucose control between babies.

**Table 3:** Comparison of glucose control and insulin delivery within feasibility and pilot study

	Accuracy (n=20)	Intervention study control (n=10)	Intervention study real-time CGM (n=10)
Sensor glucose levels			
Per cent time in range			
2.6–10 mmol/L*	78.4 (58.9–94.2)	59.0 (43.8–97.5)	76.5 (63.8–97.3)
4.00 to 8.0 mmol/L	45.7 (34.6–65.7)	33.0 (22.5–81.0)	37.0 (30.0– 51.2)
>10.0 mmol/L	21.6 (5.8–40.9)	40.0 (3.0–57.3)	23.5 (2.8–35.3)
<2.6 mmol/L	0.0 (0.0–0.0)	0.0 (0.0–0.0)	0.0 (0.0–0.0)
Mean SG (mmol/L)	8.3 (7.4–9.4)	9.5 (6.5–11.5)	8.5 (6.7–9.4)
SD of SG (mmol/L)	2.39 (1.77–3.67)	2.50 (1.46–4.32)	1.96 (1.54–3.87)
Hypoglycaemia (sensor data)†			
No of babies with > 1 episode of hypoglycaemia	2	2	1
No of episodes of hypoglycaemia	2	2	1
Length of episodes (mins)	40–70	325–410	205
Blood glucose (<2.6 mmol/L)			
No of episodes of hypoglycaemia†	3†	2	1
Insulin infused (U/kg/day)		0.02 (0.00–0.06)	0.03 (0.01–0.06)
Number of blood glucose levels per day		5.36 (3.43–5.82)	5.14 (4.29–5.43)

Greater time in target 2.6–10 mmol/L in both study groups using real-time CGM (intervention arm of the pilot study and accuracy study). Lower prevalence of hyperglycaemia in those using real-time CGM and prolonged periods of hypoglycaemia in the control arm of the pilot study. No difference in the frequency of BG monitoring or insulin infused between the arms of the pilot study. Data are presented as median (IQR).

*Primary endpoint.

Hypoglycaemic episode defined as any BG <2.6 mmol/L or SG <2.6 mmol/L>30 min. There was one episode where BG<2.6 mmol/L but the SG fell to a nadir of 2.7 mmol/L.

BG, blood glucose; CGM, continuous glucose monitoring; SG, sensor glucose.

#### Efficacy

Median (IQR) per cent time in target range 2.6–10 mmol/L was greater within the intervention than control 77% (64%–97%) versus 59% (44%–98%), respectively. The differences appeared to relate to the per cent time SG >15 mmol/L, median (IQR) 5.0 (0.0–13.8) to 0.0 (0.0–8.3) in control and intervention group, respectively. There were no differences in the rate of dextrose, amino acids, lipids, enteral feeds or total insulin infused or number of blood tests between the study groups (table 1). There was a further 2626 hours of CGM data collected from 20 babies within the feasibility study; with median (range) for individual babies of 143.75 (7.25–165.58) hours. There was a wide variability in glucose profiles, and data demonstrating the per cent time within different target thresholds are provided in table 3.

#### Safety

In the pilot study there were two clinically documented episodes of hypoglycaemia (BG <2.6 mmol/L), and in different babies in the control group, both events related to loss of central access. One of these babies had a further episode of SG <2.6 mmol/L (5 hours 45 min), but this was not observed or documented by the clinical team. In the intervention group there was one episode when the BG was documented by the clinical team to be <2.6 mmol/L but the lowest SG reading was 3.5 mmol/L, one baby had a SG <2.6 mmol/L for 3 hours 25 min, but the lowest BG at this time was 3.2 mmol/L. None of the babies were on insulin at the time, and there were no clinical concerns about hypoglycaemia.

### Staff perspective of impact on clinical care

**Figure 4:**
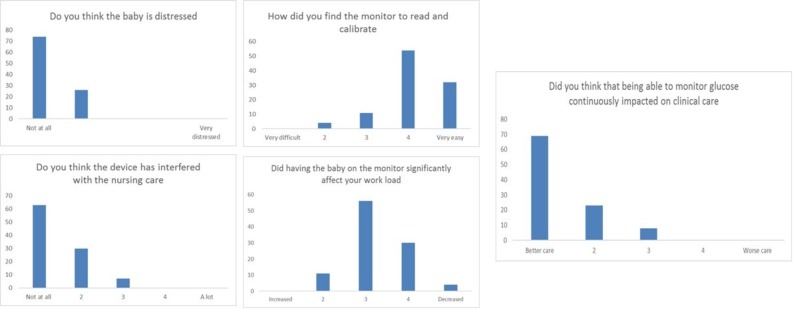
Staff assessment of the use of continuous glucose monitoring response to staff questionnaire. Data are presented as a per cent of the responses received.

Expectations varied greatly with some nurses excited about the potential to limit the need for frequent BG sampling and avoiding ‘hurting the baby’, but some had concerns about increased workload, sensor insertion and risk of ‘tissue damage’ or difficulties in positioning a baby following sensor insertion. Comments varied from ‘exciting’ to ‘How much extra time will it take up’. There were occasions with loss of connectivity between the sensor and the monitor, for example, if a baby was moved out of an incubator away from the monitor. On such occasions it was evident that the transmitter range was limited if blocked by objects between the baby and monitor. This could be resolved by moving the monitor closer to the baby. The use of both predictive and threshold alarms was found challenging by staff who felt they were an unnecessary addition to recording SG levels hourly. Frequent requests were made to silence these alarms, and subsequently they were turned off in the early stages within the studies to ensure continued staff engagement. After caring for a baby with a CGM there was an over-riding view that the intervention improved the quality of care (figure 4). Comments included ‘found the monitor to be useful when the baby was on insulin’, ‘the chart … is really useful’ ‘I think this is the best treatment’ and ‘it is not extra workload’.

## Discussion

These studies are the first to explore the utility of CGM to support targeting of glucose control with guidance on insulin delivery in preterm babies. The sensors were well tolerated in these preterm infants despite the babies’ low birth weight, limited subcutaneous tissue and potential risk of infection. The lack of data collection in two of the control babies is in keeping with problems in other study populations.^25 26^ The size and position of the transmitter was not felt to interfere with care and staff reported the intervention lead to improved care.

Our data showed the CGM to be comparable in terms of accuracy to the point-of-care devices that are currently used in clinical practice. The SG levels tracked BG levels even if typically with approximately 0.5 mmol/L difference. There were episodes when only one of SG or BG reached the threshold for hypoglycaemia, but the trend in falling CGM did prompt the measurement of BG. This difference may be related to the physiological differences between blood and interstitial glucose levels, particularly with rapidly changing BG levels. The benefits of CGM would be in guiding the need to check BG levels rather than point accuracy. This is particularly important in these infants, where BG levels are taken infrequently, and abnormalities can easily be missed.^19^ This is demonstrated by the prolonged periods of hypoglycaemia (5–6 hours), in those receiving standard care (with masked CGM data collection). In comparison, in those with real-time CGM where no episodes lasted >30 min. This is clinically important where there remains controversy regarding hypoglycaemic thresholds but length of exposure could impact on outcomes.^27^ Having used the CGM to target glucose levels we did not have sufficient values <2.6 mmol/L to report a separate mean absolute relative difference for this data.

Different strategies are used to target glucose control in the preterm infant, each with different risks and benefits. Reducing parenteral intake risks compromised nutritional delivery, while insulin infusions can lead to hypoglycaemia. A recent study in preterm infants used CGM and a computer algorithm to modify glucose intake in response to hyperglycaemia.^28^ We in contrast chose a strategy that aimed to optimise nutritional intake, with the intervention advising on insulin therapy if hyperglycaemia occurred. It is therefore unique in that it combines CGM with a guideline for the use of insulin therapy. This design is easy to adopt in clinical practice as it does not involve the need for frequent changes to parenteral nutrition, which may affect nutritional and electrolyte delivery, as well as staff workload and cost.

The study assessed the feasibility of the combined intervention to target glucose control. In the standard care group, the target remained 2.6–10 mmol/L (current standard practice), as changing the target would have required increased BG sampling and risked patient safety. To ensure clear blinding of the clinical team to the glucose levels in the control group had the iPro 2 masked CGM, which uses a different calibration algorithm compared with the Paradigm Veo and this may have introduced bias between the study groups. However, the same sensors and method for calibration were used in both study groups.^29^ In addition, there remains controversy regarding whether stochastic adjustments are required to reduce the bias in assessment of accuracy when using the same glucose values both to determine the intervention and to assess outcome. With these studies it would have been impractical to take additional BG samples and we have not performed any post hoc stochiastic adjustment,^30 31^ but the level of differences seen are unlikely to be explained by any of these factors.

The aim was not for CGM to replace BG sampling but to augment it and there was no difference in number of blood samples between study groups. Increasing confidence in the accuracy of CGM devices, and the desire to limit handling of preterm babies, may lead to reduced BG sampling in the future. The lack of benefit from predictive trend alarms was disappointing, but it may be with more experience that the alarms could be used. Study limitations include small sample size and single centre design.

Previous attempts to target glucose control with insulin in adult or neonatal intensive care have resulted in significant increases in prevalence of hypoglycaemia.^19 32^ In contrast, this study shows the use of real-time CGM provides an opportunity to track changes in glucose control in real time. This is important in providing guidance to clinical staff in a population where drug infusions as well as insulin sensitivity and secretion all change both frequently and rapidly and place babies at risk. Despite some initial concerns the nursing staff reported that the CGM improved care. These studies suggest that CGM has the potential to safely support the targeting of glucose control in the vulnerable preterm infant, reducing extreme hyperglycaemic and clinically silent hypoglycaemic exposure. Larger studies are required to validate these findings and to address the question of optimal targets in relation to clinical impact and how best to achieve them.
