# Supplementary figures and images for: Targeting glucose control in preterm infants: pilot studies of continuous glucose monitoring
Source: Arch Dis Child Fetal Neonatal Ed. 2018 Sep 19;104(4):F353–9. doi: 10.1136/archdischild-2018-314814 (PMC6764251; doi:10.1136/archdischild-2018-314814)

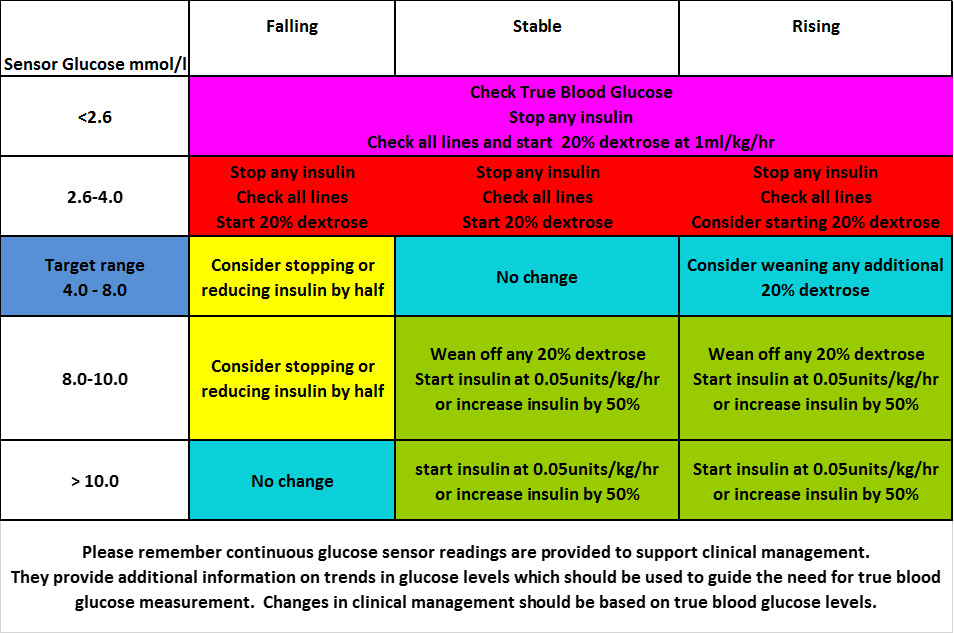

Supplement: Supplementary file 1 [file fetalneonatal-2018-314814supp001.jpg]
